# Identification of molecular subtypes of dementia by using blood-proteins interaction-aware graph propagational network

**DOI:** 10.1093/bib/bbae428

**Published:** 2024-09-03

**Authors:** Sunghong Park, Chang Hyung Hong, Sang Joon Son, Hyun Woong Roh, Doyoon Kim, Hyunjung Shin, Hyun Goo Woo

**Affiliations:** Department of Physiology, Ajou University School of Medicine, Worldcup-ro 164, Yeongtong-gu, Suwon, 16499, Republic of Korea; Department of Psychiatry, Ajou University School of Medicine, Woldcup-ro 164, Yeongtong-gu, Suwon, 16499, Republic of Korea; Department of Psychiatry, Ajou University School of Medicine, Woldcup-ro 164, Yeongtong-gu, Suwon, 16499, Republic of Korea; Department of Psychiatry, Ajou University School of Medicine, Woldcup-ro 164, Yeongtong-gu, Suwon, 16499, Republic of Korea; Department of Physiology, Ajou University School of Medicine, Worldcup-ro 164, Yeongtong-gu, Suwon, 16499, Republic of Korea; Department of Biomedical Science, Graduate School, Ajou University, Worldcup-ro 164, Yeongtong-gu, Suwon, 16499, Republic of Korea; Department of Industrial Engineering, Ajou University, Worldcup-ro 206, Yeongtong-gu, Suwon, 16499, Republic of Korea; Department of Artificial Intelligence, Ajou University, Worldcup-ro 206, Yeongtong-gu, Suwon, 16499, Republic of Korea; Department of Physiology, Ajou University School of Medicine, Worldcup-ro 164, Yeongtong-gu, Suwon, 16499, Republic of Korea; Department of Biomedical Science, Graduate School, Ajou University, Worldcup-ro 164, Yeongtong-gu, Suwon, 16499, Republic of Korea; Ajou Translational Omics Center (ATOC), Research Institute for Innovative Medicine, Ajou University Medical Center, Worldcup-ro 164, Yeongtong-gu, Suwon, 16499, Republic of Korea

**Keywords:** dementia subtype diagnosis, Alzheimer’s disease, vascular dementia, plasma protein biomarker, protein–protein interaction, graph neural network

## Abstract

Plasma protein biomarkers have been considered promising tools for diagnosing dementia subtypes due to their low variability, cost-effectiveness, and minimal invasiveness in diagnostic procedures. Machine learning (ML) methods have been applied to enhance accuracy of the biomarker discovery. However, previous ML-based studies often overlook interactions between proteins, which are crucial in complex disorders like dementia. While protein–protein interactions (PPIs) have been used in network models, these models often fail to fully capture the diverse properties of PPIs due to their local awareness. This drawback increases the chance of neglecting critical components and magnifying the impact of noisy interactions. In this study, we propose a novel graph-based ML model for dementia subtype diagnosis, the graph propagational network (GPN). By propagating the independent effect of plasma proteins on PPI network, the GPN extracts the globally interactive effects between proteins. Experimental results showed that the interactive effect between proteins yielded to further clarify the differences between dementia subtype groups and contributed to the performance improvement where the GPN outperformed existing methods by 10.4% on average.

## Introduction

Dementia is characterized by cognitive decline and impairment in daily living functions due to degenerative brain changes, which is subdivided into several subtypes according to pathophysiological characteristics [1, 2], the most common being Alzheimer’s disease (AD), followed by vascular dementia (VD). The diagnoses of dementia subtypes are based on cognitive function assessment, neuroimaging such as positron emission tomography (PET) and magnetic resonance imaging (MRI), and cerebrospinal fluid (CSF) biomarkers [3–7]. First, cognitive function assessment is an essential process for diagnosing dementia, measured by a standardized neuropsychological test, but it may vary depending on the patient’s condition on the day of testing and can also vary based on the examiner. Second, neuroimaging provides objective evidence on the deposition of amyloid beta (Aβ) or the detection of tau tracer in PET and the determination of hippocampal or medial temporal neurodegeneration in MRI, but requires high costs [6]. Third, CSF biomarkers, such as Aβ_42_, total tau (T-tau), and phosphorylated tau (P-tau), can be utilized; however, patients are required to undergo bed rest for several hours and, although rare, potentially fatal side effects can occur. Hence, a diagnostic marker for differentiating the possible pathophysiology and subtypes of dementia with high reliability, cost-effectiveness, and minimal invasiveness is required for clinical practicality.

Recently, the discovery of blood biomarkers for dementia has raised the possibility of low-variable, low-cost, and less-invasive alternative methods for assessing the possible pathophysiology and diagnosis of dementia subtypes [8–10]. Moreover, the application of machine learning (ML) has enabled the discovery of more sophisticated dementia-associated biomarkers and the prediction of target outcomes with high accuracy [11–14]. However, most existing studies only considered the independent effects of proteins excluding the interactive effects between proteins. This approach overlooks the fact that multiple proteins with small effect sizes collectively contribute to the phenotype owing to their interactions. Protein interaction–based disease-related biomarkers, which capture the complex interplay between proteins in disease, are of great importance for understanding molecular pathogenesis, risk assessment, and disease classification [15]. With the development of high-throughput technologies, protein–protein interaction data have grown rapidly to cover nearly the entire proteome [16], and, at the same time, network-based methods using protein interactions have become prominent as ML techniques can be applied to identify sophisticated biomarkers [17]. Dementia is a complex disorder involving the interactions between specific molecular pathways, making the effects of protein interactions more important [18, 19]. Therefore, a model that considers protein interactions within molecular pathways is needed.

A graph convolutional network (GCN) has utilized protein–protein interactions (PPIs) among blood proteins could have unveiled distinct dementia subtypes [20]. In the GCN, the feature of each node is aggregated with the features of adjacent nodes through edges by a graph convolutional layer. This process is applied to the PPI network in which the nodes, edges, and features correspond to the proteins, PPIs, and expression values by blood sequencing, respectively. Therefrom, the extracted protein features represent the interactive effect between proteins in that the expression value of each protein is aggregated with the expression values of adjacent proteins on the PPI network. This approach of representing the protein interaction by applying the GCN to the PPI network is used for various target tasks in addition to novel PPI identification [21–23], disease type classification [24], and cancer survival prediction [25].

When analyzing protein interactions, it is crucial that the effects of indirect interactions among proteins also significantly contribute to disease progression. Proteins transmit signals to other proteins at a distance and collectively have an impact on the progression of certain diseases. This mechanism suggests that the application of ML to the PPI network should perform targeted tasks through the global awareness of protein interactions, which extracts the full range of interaction effects by considering the whole PPI network. However, the graph convolutional operation of GCN is limited to the local awareness of PPIs. In other words, it performs feature aggregation solely between 1-hop neighboring nodes, thus restricting its ability to capture only local interactions between directly connected proteins within the PPI network. To overcome this limitation, extended GCN can be employed to aggregate features from multi-hop neighbors.

Previously, two main approaches have been employed to extend GCN for *K*-order feature aggregation: ‘multiplied convolution filter’ and ‘parallelized network architecture’. The multiplied convolution filter–based approach repeatedly multiplies the normalized adjacent matrix *K* times so that the graph convolution filter is represented to *K*-th powered single matrix. This approach started from Simple Graph Convolution (SGC) [26] which is empowered by Exponential Graph Convolution (EGC) and Linear Graph Convolution (LGC) [27]. In GCN, *K* graph convolutional layers are required for feature aggregation with *K*-hop neighboring nodes, but SGC is simplified to a single layer through a *K*-th-powered graph convolution filter. EGC derives the graph convolution filter by combining the graph Laplacian up to the *K*-th power with the coefficients of the exponential power series. LGC is the graph convolution filter by linear combination of graph Laplacian monomials up to *K*-th power.

Next, the parallelized network architecture consists of *K* number of graph convolution filters up to *K*-hop neighboring nodes on the adjacent matrix. Each filter is applied to parallel graph convolutional layers to individually aggregate node features. The representative method of this approach is MixHop [28] proposing the higher-order graph convolutional architecture, which is further developed as universal GCN (UGCN) [29] and mixed-order GCN (MOGCN) [30]. The three methods reveal clear differences in how they merge node features extracted from parallel graph convolution layers. To merge multiple feature sets, the simple concatenation, the attention mechanism, and the ensemble module are utilized by MixHop, UGCN, and MOGCN, respectively.

These extended models of GCN can reflect a wider range of PPI, but they do not consider the entire properties of PPI network. Thus, their capacity is constrained to reflecting PPI within a limited local range, leading to several following issues: (1) *Neglecting the structural attributes of PPI network*: PPI network is a complex network with a hierarchical structure, including subnetworks. Focusing solely on interactions between neighboring proteins might overlook critical features, modules, and clusters within the entire network [31, 32]. (2) *Missing key components of PPI network*: hub proteins situated in specific regions of PPI network and the connections between them exert a significant influence on PPI across the entire network. However, these critical elements may not always be accounted for in local PPI analyses [33]. (3) *Emphasizing the noisy interactions within the PPI network*: as the PPI network comprises PPI data from diverse sources, it inherently incorporates experimentally noisy interactions. Utilizing local PPI approaches poses the risk of overestimating these noisy interactions [34–36]. While extending the GCN-extended model structure and broadening its configuration may mitigate these issues to some extent, it remains a temporary solution incapable of achieving global PPI representation. This limitation stems from graph convolution’s reliance on locality, facilitating feature aggregation between adjacent nodes. To address these challenges comprehensively and accurately capture the properties of PPI networks, graph neural networks necessitate feature aggregation based on globality.

In this study, we introduce a novel graph neural network termed ‘graph propagational network’ (GPN). Central to our approach is the graph propagation layer, which generates a globally aggregated feature representation by spreading the features of each node across all nodes within the graph. This enables the diagnosis of dementia subtypes based on blood biomarkers, leveraging the interactions among blood proteins to accurately reflect the key components and structural properties of the PPI network.

### Overview of the study

**Figure 1:**
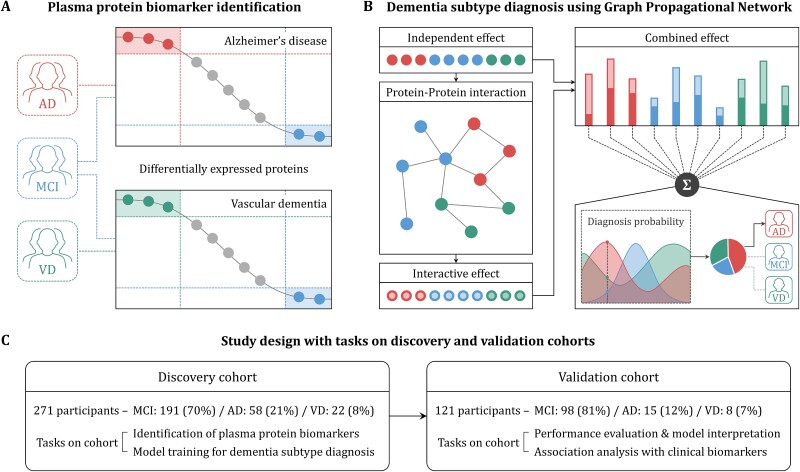
Schematic overview of the study design. The analysis strategy consists of two stages: plasma protein biomarker identification (A) and dementia subtype diagnosis using the GPN (B). In the first stage, the expression levels of proteins in participants with MCI are compared to those in participants with AD and VD, respectively. Therefrom, proteins showing significant differences between diagnosis groups are identified as biomarkers. In the second stage, the proposed model performs dementia subtype diagnosis utilizing the identified biomarkers. Our model not only takes into account the expression value of each plasma protein biomarker (independent effect) but also considers the effects resulting from interactions between proteins (interactive effect) for diagnosing dementia subtypes. In this study, discovery and validation cohorts are configured for independent evaluation of the proposed method (C). The discovery cohort is applied to identify plasma protein biomarkers and train the proposed model for dementia subtype diagnosis. The validation cohort is utilized for performance evaluation, model interpretation, and association analysis with clinical biomarkers.

This study comprises two main stages. Firstly, in the plasma protein biomarker identification stage (Fig. 1A), the expression levels of plasma proteins in participants with mild cognitive impairment (MCI) are compared to those in participants with AD and VD. Proteins exhibiting significant differences between participant groups are then identified as plasma protein biomarkers for dementia subtypes. Subsequently, the proposed method, GPN, is employed to classify MCI, AD, and VD based on the identified protein biomarkers (Fig. 1B). The GPN not only considers the expression values of individual proteins but also accounts for the effects resulting from interactions between proteins. In addition, to objectively evaluate our method, we established distinct discovery and validation cohorts (Fig. 1C). In the discovery cohort, we identified plasma protein biomarkers and trained the dementia subtype diagnosis model. On the other hand, the validation cohort was employed to assess the performance of the model and conducted various analyses regarding the interpretation and utilization of the model’s outcomes.

## Methods

### Plasma protein biomarker identification

#### Study participants

Participants were from the Biobank Innovations for chronic Cerebrovascular disease With ALZheimer’s disease Study (BICWALZS) [37] at Ajou University Hospital (Suwon, Republic of Korea), a disease-focused biobank supported and funded by the National Institute of Health of the Korea Disease Control and Prevention Agency. The participants were diagnosed with MCI, AD, and VD based on he expanded Mayo Clinic criteria [38], the core clinical criteria proposed by the National Institute on Aging and the Alzheimer’s Association working group in 2011 [5], and the major vascular neurocognitive disorder criteria proposed in the fifth edition of the Diagnostic and Statistical Manual of Mental Disorders [39], respectively. Therefrom, the 392 participants were categorized into 289 MCI (74%), 73 AD (19%), and 30 VD (8%) participants. Additionally, the participants were divided into the discovery and validation cohorts based on the time of recruitment. The discovery cohort included 271 participants recruited between November 2016 and August 2019. Of these, 191 (70%), 58 (21%), and 22 (8%) had MCI, AD, and VD, respectively. The validation cohort included 121 participants, recruited from September 2019 to September 2020, consisting of 98 (81%) participants with MCI, 15 (12%) with AD, and 8 (7%) with VD.

#### Olink proteomic assays

Plasma samples from participants are profiled by the Olink Proteomics using proximity extension assay (PEA) technology. The Olink Neurology panel containing 92 proteins is assayed in this study, where the assay of the neurology panel includes the established markers associated with neurobiological processes and neurological diseases (e.g. neurodevelopment, axon guidance, synaptic function, or specific conditions such as AD) [40]. Quality control of the raw data was performed by using the internal and external controls in the panels, and the protein expression levels were normalized by the interplate control normalization.

#### Gene Ontology analysis

Gene Ontology (GO) analysis on the selected plasma proteins is conducted to investigate functional annotations and understand the biological meaning of the biomarkers. GO analysis is performed by using Bioinformatics Resources provided by the Database for Annotation, Visualization, and Integrated Discovery (DAVID, https://david.ncifcrf.gov, version 2021) [41, 42].

### Dementia subtype diagnosis using graph propagational network

**Figure 2:**
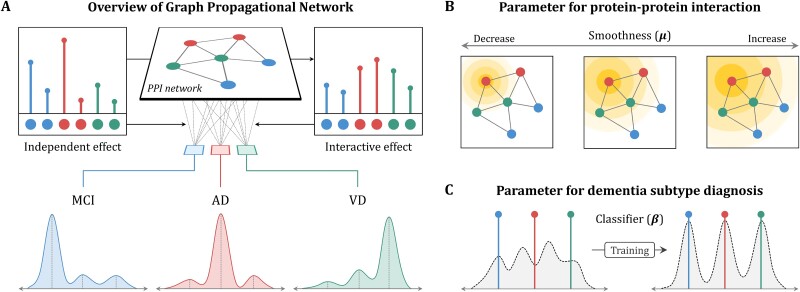
Schematic description for GPN. Our model extracts the interactive effect by propagating the independent effect to the PPI network, combines both effects, and diagnoses dementia subtypes (A). GPN consists of two parameters: smoothness and classifier. Smoothness controls the extent of propagating the independent effect (B). Decreasing smoothness highlights lower-order PPIs, while increasing smoothness takes higher-order PPIs important as well. Classifier indicates the coefficient matrix, which is applied to the combined effect, for dementia subtype diagnosis (C).

The proposed model, GPN, aims to capture not only the effects of protein itself but also the interactive effects between proteins on the classification of dementia subtypes (Fig. 2A). To extract the interactive effect by propagating the independent effects across the network, the GPN was applied to the PPI network obtained from the STRING database (https://string-db.org/) [43, 44] encompassing the global interactions between proteins. During this process, the smoothness parameter controls the range of PPIs determining the extent to which independent effect is propagated within the GPN (Fig. 2B), where the smoothness of the PPI network indicates that the expression values of the interacting proteins are similarly represented to each other. As the smoothness decreases, lower-order PPIs are considered to be more important in GPN, highlighting interactions between nearby proteins. Vice versa, when the smoothness increases, higher-order PPIs, reflecting interactions between distant proteins, become increasingly more influential in GPN. The smoothness is usually a predefined parameter, but in GPN, it is a learnable parameter that is optimized through the model training. After that, GPN combines the extracted interactive effects with the independent effect and diagnoses dementia subtypes by the classifier (Fig. 2C).

#### Formulation

The data matrices for the independent effect and PPI network are denoted as $\mathbf{X}\in{\mathbb{R}}^{{p}\times{n}}$ and $\mathbf{W}\in{\mathbb{R}}^{{p}\times{p}}$, respectively, where $p$ is the number of plasma protein biomarkers and $n$ is the number of participants. The interactive effect, indicated by $\mathbf{F}$, is extracted by propagating the independent effect to the PPI network, and in this process, GPN aims to reflect the global interactions among proteins. For this purpose, graph-based semi-supervised learning [45–47] is applied to the proposed method, and therefrom, the objective function for $\mathbf{F}$ is defined as follows:


(1) \begin{equation*} \underset{\mathrm{F}}{\min }\ {\left(\mathbf{F}-\mathbf{X}\right)}^{\mathrm{T}}\left(\mathbf{F}-\mathbf{X}\right)+\mu{\mathbf{F}}^{\mathrm{T}}\mathbf{LF} \end{equation*}

where $\mathbf{L}$ is the graph Laplacian, defined as $\mathbf{L}=\operatorname{diag}\left(\mathbf{W}\right)-\mathbf{W}$, and $\mu$ is a trainable parameter that trades off the loss (the first term) and the smoothness (the second term). The solution of equation (1) is obtained in the closed form as follows:


$${\displaystyle \begin{array}{c}\mathbf{F}={\left(\mathbf{I}+{\mu} \mathbf{L}\right)}^{-1}\mathbf{X}={\boldsymbol{\Phi}}^{-1}\mathbf{X}\in{\mathbb{R}}^{{p}\times{n}}.\end{array}}$$

where $\left(\mathbf{I}+{\mu} \mathbf{L}\right)$ is replaced with $\boldsymbol{\Phi}$ for the brief formulation.

Next, the GPN combines the extracted interactive effect $\mathbf{F}$ with the independent effect $\mathbf{X}$. Given the combining coefficient denoted as ${\alpha}$, to prevent the problem of combining coefficient turning negative during the model training, ${\alpha}$ is transformed into a probability form as


$${\theta} =\frac{{1}}{{1}+{{e}}^{-{\alpha}}}.$$

Therefrom, the combined effect, represented as $\mathbf{Z}\in{\mathbb{R}}^{{p}\times{n}}$, is derived by a linear combination of $\mathbf{X}$ and $\mathbf{F}$ as follows:


$$\mathbf{Z}={\theta} \mathbf{F}+\left(1-{\theta} \right)\mathbf{X}$$

Then, the combined effect $\mathbf{Z}$ is multiplied by the classifier parameter denoted as $\boldsymbol{\mathrm{\beta}} \in{\mathbb{R}}^{{p}\times{c}}$, where ${c}$ is the number of dementia subtypes. Finally, the individual diagnosis, represented as probability for each dementia subtype, is obtained by using the softmax function.


$$\mathbf{P}=\mathrm{softmax}\left({\boldsymbol{\mathrm{\beta}}}^{\mathrm{T}}\mathbf{Z}\right)\in{\mathbb{R}}^{{c}\times{n}}$$

#### Optimization

The objective function for GPN is formulated as follows:


(2) \begin{equation*} {\displaystyle \begin{array}{c}\underset{{\alpha}, {\mu}, \boldsymbol{\mathrm{\beta}}}{\mathrm{argmin}}\ {\mathcal{L}}+{\delta} {\mathcal{R}}\end{array}} \end{equation*}

where ${\mathcal{L}}$ and ${\mathcal{R}}$ indicate the cross-entropy loss and the regularization term, respectively, and ${\delta}$ is the combining coefficient $\left({\delta} >{0}\right)$. The cross-entropy loss ${\mathcal{L}}$ and the regularization term ${\mathcal{R}}$ in (2) defined as


$${\mathcal{L}}=-\frac{{1}}{{n}}\mathrm{Tr}\left({\mathbf{Y}}^{\mathrm{T}}\mathbf{\log}\,\mathbf{P}\right),\quad{\mathcal{R}}={\left\Vert{\alpha} \right\Vert}_{{2}}^{{2}}+{\left\Vert{\mu} \right\Vert}_{{2}}^{{2}}+{\left\Vert{\boldsymbol{\beta}} \right\Vert}_{{2}}^{{2}}$$

where $\mathbf{Y}\in{\mathbb{R}}^{{c}\times{n}}$ is the labeled data indicating the real diagnosis for dementia subtypes. The objective function is optimized by gradient descent method [48–50]. First, the gradient *w.r.t.*, the coefficient matrix $\boldsymbol{\mathrm{\beta}}$ is derived as


$${\displaystyle \begin{array}{c}\nabla\boldsymbol{\mathrm{\beta}} =\frac{{1}}{{n}}\mathbf{Z}{\left(\mathbf{P}-\mathbf{Y}\right)}^{\mathrm{T}}+{2}{\delta } \boldsymbol{\mathrm{\beta}} .\end{array}}$$

Second, to find the gradient *w.r.t.*${\alpha}$, the derivative of $\mathbf{Z}$*w.r.t.*${\alpha}$ is firstly obtained. Since ${\alpha}$ has been converted to ${\theta}$, ${\partial}\mathbf{Z}/{\partial{\alpha}}$ is derived by combining the derivative of $\mathbf{Z}$*w.r.t.*${\theta}$$\left({\partial}\mathbf{Z}/{\partial{\theta}}=\mathbf{F}-\mathbf{X}\right)$ and the derivative of ${\theta}$*w.r.t.*${\alpha}$$\left({\partial{\theta}}/{\partial{\alpha}}={\theta} \left({1}-{\theta} \right)\right)$ as follows:


(3) \begin{equation*} {\displaystyle \begin{array}{c}\frac{\mathrm{\partial \mathbf{Z}}}{\partial \alpha }=\theta \left(1-\theta \right)\left(\mathbf{F}-\mathbf{X}\right)\end{array}} \end{equation*}

Then, the gradient *w.r.t.*$\alpha$ is obtained by combining $\partial \mathcal{L}/\partial \mathbf{Z}$ with (3) as


$${\displaystyle \begin{array}{c}\nabla\alpha =\frac{1}{n}\theta \left(1-\theta \right)\mathrm{Tr}\left({\left(\mathbf{F}-\mathbf{X}\right)}^{\mathrm{T}}\left(\boldsymbol{\beta} \left(\mathbf{P}-\mathbf{Y}\right)\right)\right)+2\delta \alpha .\end{array}}$$

Third, to find the gradient *w.r.t.*$\mu$, the derivative of ${\boldsymbol\Phi}^{-1}$*w.r.t.*$\mu$ is firstly represented according to the differentiation of inverse matrix as follows:


$$ {\displaystyle \begin{array}{c}\frac{\partial{\boldsymbol\Phi}^{-1}}{\partial \mu }=-{\boldsymbol\Phi}^{-1}\frac{\mathrm{\partial \boldsymbol\Phi }}{\partial \mu }{\boldsymbol\Phi}^{-1}=-{\boldsymbol\Phi}^{-1}\mathbf{L}{\boldsymbol\Phi}^{-1}.\end{array}} $$

Then, $\partial\mathbf{F}/\partial \mu$ is obtained by multiplying $\partial\mathbf{F}/\mathrm{\partial \boldsymbol\Phi }$ and $\mathrm{\partial \boldsymbol\Phi }/\partial \mu$, and since $\partial\mathbf{F}/{\partial \boldsymbol\Phi }=\mathbf{X}$, the gradient *w.r.t.*$\mu$ is derived as


$${\displaystyle \begin{array}{c}\nabla\mu =-\frac{\theta }{n}\mathrm{Tr}\left({\left({\boldsymbol\Phi}^{-1}\mathbf{L}{\boldsymbol\Phi}^{-1}\mathbf{X}\right)}^{\mathrm{T}}\left({\boldsymbol\beta} \left(\mathbf{P}-\mathbf{Y}\right)\right)\right)+2\delta \mu .\end{array}}$$

## Results

### Demographic and clinical characteristics of participants

We analyzed the data from discovery cohort (*n* = 271) and validation cohort (*n* = 121). Clinical characteristics of the participants are summarized in Table 1. The median (IQR) age was 73 (67–77) years, the Montgomery–Asberg Depression Rating Scale (MADRS) was 14 (6–24), the Mini-Mental State Examination (MMSE) was 24 (21–27), the Clinical Dementia Rating Sum of Boxes (CDR-SB) was 2.0 (1.5–4.0), and the Global Deterioration Scale (GDS) was 3 (3–4). Of the 392 participants, 71.2% were female; 13.5% and 27.0% had APOE *ε*2 and *ε*4 carriers, respectively; and the positive ratio for Aβ, medial temporal lobe atrophy (MTA), and white matter hyperintensity (WMH) were 29.8%, 22.8%, and 37.4%, respectively. There were no differences between the discovery and validation cohorts in any other characteristic, except for MTA-positive. Furthermore, we divided the participants in each cohort according to their diagnosis and compared the demographic and clinical characteristics between the subgroups (Supplementary Tables S1 and S2). In brief, significant differences were found between the subgroups in terms of major clinical characteristics.

**Table 1:** Demographic and clinical characteristics of study participants.

Characteristics	Study participants (*n* = 392)	Discovery cohort (*n* = 271)	Validation cohort (*n* = 121)	*P*-value
Age, median (IQR), yr	73 (67–77)	73 (65–77)	73 (67–77)	0.897
Female, No. (%)	279 (71.2)	192 (70.8)	87 (71.9)	0.927
MADRS, median (IQR)	14 (6–24)	14 (6–24)	15 (4–24)	0.995
MMSE, median (IQR)	24 (21–27)	24 (21–27)	24 (20–26)	0.717
CDR-SB, median (IQR)	2.0 (1.5–4.0)	2.0 (1.5–4.0)	2.0 (1.5–3.0)	0.166
GDS, median (IQR)	3 (3–4)	3 (3–4)	3 (3–4)	0.313
APOE genotype, No. (%)				
*ε*2 allele carrier	53 (13.5)	38 (14.0)	15 (12.4)	0.536
*ε*4 allele carrier	106 (27.0)	73 (26.9)	33 (27.3)	0.921
Aβ-positive, No. (%)	117 (29.8)	91 (33.6)	26 (21.5)	0.086
MTA-positive^a^, No. (%)	89 (22.8)	70 (26.0)	19 (15.7)	0.022
WMH-positive^b^, No. (%)	146 (37.4)	104 (38.7)	42 (34.7)	0.809

Abbreviations: IQR, interquartile range; MADRS, Montgomery–Asberg depression rating scale; MMSE, Mini-Mental Status Examination; CDR-SB, Clinical Dementia Rating Sum of Boxes; GDS, Global Deterioration Scale; APOE, apolipoprotein E; Aβ, amyloid beta; MTA, medial temporal lobe atrophy score; WMH, white matter hyperintensity.

a MTA scale is divided into left and right and subdivided into 0–4 according to severity, and in this study, MTA-positive was set for cases where the sum of left and right sides was 5 or more.

b WMH scale is divided into three types (mild, moderate, and severe), and WMH-positive was set for moderate and severe.

### Differentially expressed plasma proteins

**Figure 3:**
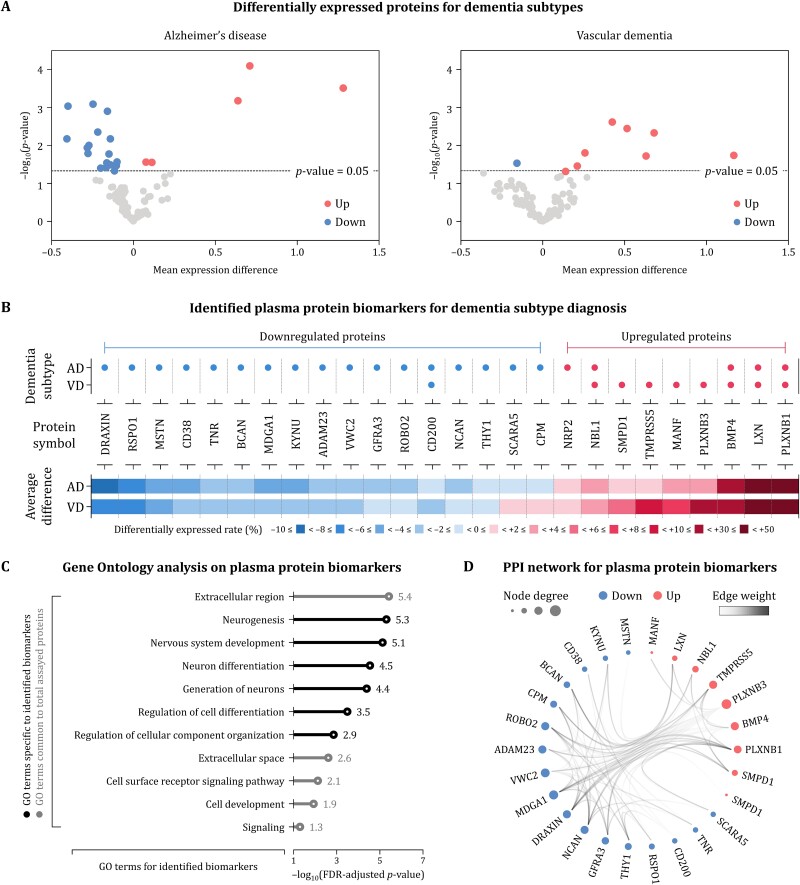
Results for plasma protein biomarkers identification. Of the 90 assayed plasma proteins, 22 and 9 proteins were differentially expressed in AD and VD, respectively (A). For dementia subtype diagnosis, 9 upregulated and 17 downregulated proteins were identified as plasma protein biomarkers (B). GO analysis revealed that 11 GO terms were significant for the 26 plasma protein biomarkers, where five terms were common to the total 90 proteins and six terms were specific to the identified biomarkers (C). The interaction network for the identified biomarkers was constructed based on the entire PPI (D). The averages of node degrees for upregulated and downregulated proteins were almost similar, and both the number and weight of intragroup edges were observed to be greater than those of intergroup edges.

Plasma protein levels of the participants were profiled using PEA, identifying the differentially expressed proteins in AD and VD compared to those in MCI. A total of 90 proteins among 92 proteins in the panel could be detected after applying cutoff of the missing frequencies above 75%. We identified 22 and 9 proteins that were differentially expressed in participants with AD and VD, respectively, including 5 common proteins (Fig. 3A; Supplementary Tables S3 and S4). The significance of the subtype difference was estimated by the linear regression analysis using ‘limma’ [51] implemented in an R/Bioconductor (https://www.bioconductor.org/). As a result, 26 proteins were identified as plasma protein biomarkers for dementia subtype diagnosis, consisting of 9 and 17 significantly upregulated and downregulated proteins (Fig. 3B). The common biomarkers for AD and VD showed an average of fold difference greater than five times higher than that of other biomarkers (Supplementary Table S5).

Next, we performed GO analysis to identify enriched functional categories among the identified plasma protein biomarkers. We found 11 significant GO terms for the identified 26 biomarkers [FDR-adjusted *P*-values (*Q*-values) <.05, with coverage ratios >40%]. Comparing with GO analysis on the total 90 proteins, there were five common GO terms and six GO terms specific to the identified biomarkers (Fig. 3C; Supplementary Table S6). The former were generally related to the cellular anatomical entity outside plasma membranes and the molecular signal transmission within a biological system, while the latter were mainly associated with the generation and progression of cells and tissues in the nervous system. The significance level of the GO terms specific to the identified biomarkers was on average 1.6 times higher than that of the common GO term.

Furthermore, we constructed the interaction network for the identified plasma protein biomarkers from the entire PPI (Fig. 3D). The constructed network included a total of 79 interactions, showing a density of 24.31%. TMPRSS5, an upregulated protein, showed the highest node degree by interacting with seven other biomarkers, followed by MDGA1, a downregulated protein, which related to six biomarkers. The average of node degrees for upregulated and downregulated proteins were almost similar, 3.00 and 3.06, respectively. Additionally, more interactions were observed between proteins within the same group, with 43 intragroup interactions and 36 intergroup interactions. The average of edge weights for intragroup interactions was 0.33, which was 31.02% higher than that of 0.25 for intergroup interactions.

### Performance evaluation

#### Experimental settings

The performance of GPN for dementia subtype classification was evaluated by comparing those of seven different methods: GCN [20], SGC [26], EGC [27], LGC [27], MixHop [28], UGCN [29], and MOGCN [30]. For GPN, the smoothness parameter $\mu$ was variated in the range of $\left\{{10}^{-2},{10}^{-1},1,{10}^1,{10}^2\right\}$, and the combining coefficient $\alpha$ was set to 0. The model architecture of the comparison methods was constructed with reference to the best performance reported in each paper. Therefrom, the maximum order of interaction between proteins that reflected in those methods were 2 for GCN and SGC, 3 for EGC, 4 for MixHop and UGCN, 5 for LGC, and 6 for MOGCN. All models, including GPN, were trained using the ADAM optimizer [52] with a learning rate of 0.001, and the performance was measured by area under the receiving operating characteristic curve (AUROC), and area under the receiving precision–recall curve (AUPRC).

#### Performance comparison

**Figure 4:**
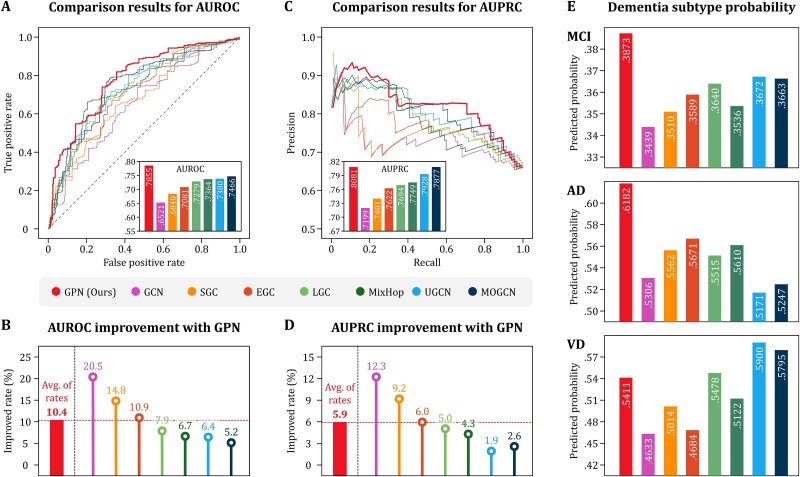
Performance comparison results. The AUROC performance of GPN was compared with that of seven existing methods (A). GPN showed the best performance, with an average of 10.4% higher than the comparison methods (B). The AUPRC performance was also compared (C), and GPN again derived the best result, with an average of 5.9% higher than others (D). Comparing the average predicted probabilities of dementia subtypes for each diagnostic group, GPN made the most distinctive diagnoses for MCI and AD and slightly clearer for VD (E).

At first, the AUROC performance of all methods ranged from 0.65 to 0.79, with an average performance of 0.7223 (Fig. 4A). Of those, the GPN performed the most accurate diagnosis with an AUROC of 0.7855, and, as a baseline method, GCN showed the lowest performance with an AUROC of 0.6521. The multiplied convolution filter–based methods, SGC, EGC, and LGC, showed AUROC performance of 0.6840, 0.7081, and 0.7279, respectively, with an average of 0.7067. On the other hand, the parallelized network architecture–based methods, MixHop, UGCN, and MOGCN, showed AUROC performance of 0.7364, 0.7380, and 0.7466, respectively, with an average of 0.7403, which is 4.76% better than that of the former methods. The GPN indicated an average of 10.4% improved AUROC performance compared to other methods (Fig. 4B). The AUROC improvement by GPN was more pronounced in methods reflecting low-order interactions. These results imply a tendency for the maximum order of interaction and performance to be proportional and suggest that the GPN was able to produce the best results by reflecting global interaction. The characteristics in the AUROC comparison results are also confirmed in the AUPRC comparison results (Fig. 4C and D). Additionally, we compared the average values of individual probabilities for dementia subtypes by the corresponding diagnostic groups (Fig. 4E). The GPN yielded the highest probability on average and more clearly predicted MCI and AD participants. For VD diagnosis, although GPN did not show the highest probability, its result was 3% higher than the average.

#### Empirical analysis

**Figure 5:**
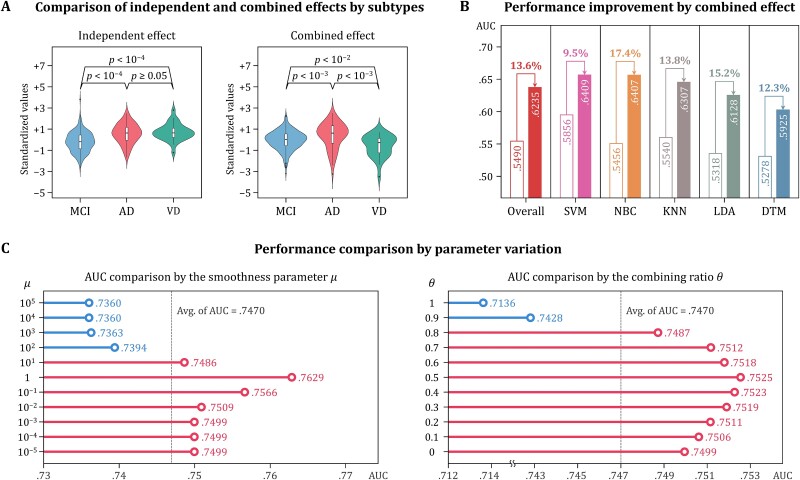
Results for empirical analysis on the discriminative power of GPN. Individual average values of protein biomarkers for the independent and combined effects were compared by dementia subtype (A). The combined effect by GPN showed significant differences between all subtypes, whereas the difference between AD and VD groups was ambiguous in the independent effects. The discriminative power of the combined effect was tested by using five traditional machine learning algorithms (B). All algorithms performed better when training the combined effect rather than the independent effect. Based on the performance comparison results according to parameter variation (C), it was observed that the discriminative power of GPN is maximized when the interactive effect derived by the smoothness near 1 is reflected in the combined effect with a similar proportion to the independent effect.

We further conducted empirical analysis on the discriminative power of GPN. At first, the difference between the independent effect $\mathbf{X}$ with the combined effect $\mathbf{Z}$ according to dementia subtypes was investigated. The individual average values of the protein biomarkers for each effect were calculated and compared by dementia subtypes (Fig. 5A). The most pronouncing feature between the two effects was the difference between AD and VD groups. In the independent effect, the difference between AD and VD groups was not significant. On the other hand, the combined effect indicated the significant difference between the two groups with *P*-value under 10^−3^. We also tested the discriminative power of the combined effect in five traditional ML algorithms: Support Vector Machines (SVM), Naïve Bayes Classifier (NBC), *K*-Nearest Neighbor (KNN), Linear Discriminant Analysis (LDA), and Decision Tree Model (DTM). The average performance of the five algorithms when training the combined effect was AUC 0.6235, which was 13.6% higher than the average performance of AUC 0.5490 when training the independent effect (Fig. 5B). As a result, the combined effect by GPN indicates that there is a significant difference between all dementia subtype groups, and this discriminative power importantly contributes to dementia subtype diagnosis using other algorithms.

Next, we observed the performance changes of GPN depending on the parameter variation (Fig. 5C). The smoothness parameter 𝜇 was set to powers of 10 from 10^−5^ to 10^5^, the combining ratio θ ranged from 0 to 1 in increments of 0.1. Therefrom, both parameters were variated to 11 levels, and the average AUC of each parameter level was compared. As the smoothness increasing, the performance remained almost unchanged until 10^−3^, then increased slightly at 10^−2^, showing the maximum AUC at 1, and decreased sharply from 10^1^ and continued to decline until 10^5^. In the case of combining ratio, performance gradually improved until the level reached 0.5, after which the AUC gradually decreased and then sharply declined from 0.8. The performance according to changes in the two parameters showed a similar pattern in which the AUC gradually increased until the two parameters reached a certain level, and then, the AUC rapidly decreased. Consequently, the combined effect derived by the smoothness and combining ratio around 1 and 0.5, respectively, is most advantageous for exploiting interactions between proteins.

### Evaluation of the functional significance of the identified biomarkers

Functional significance of the identified biomarkers was evaluated by employing statistical and explanatory analyses, respectively. In statistical analysis, we examined the aspects of changes in values for proteins and the significance of differences between dementia subtype groups. In explanatory analysis, the importance and impact on GPN of each protein were investigated by using the SHapley Additive exPlanations (SHAP) [53].

#### Statistical interpretation

**Figure 6:**
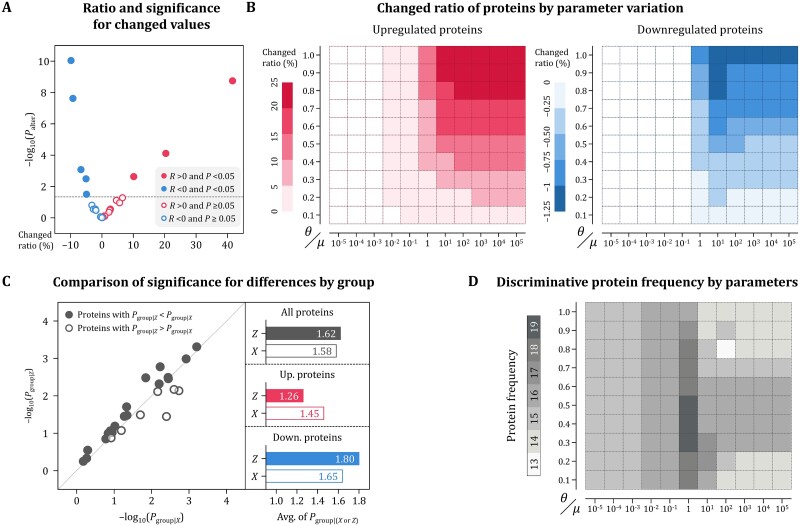
Statistical analysis results for GPN interpretation. The difference between the independent and combined effects was compared by calculating the change in protein values and its significance (A), including changing patterns for the values of upregulated and downregulated proteins by variating parameters in GPN (B). Additionally, the two effects were compared by examining the significance of the difference between diagnostic groups for each protein (C). The frequencies of proteins in the combined effect indicating significant differences between dementia subtypes were also analyzed through the parameter variation (D).

At first, the changed ratios of protein values and those significance were derived by comparing the combined effect with the independent effect (Fig. 6A). The average ratio of change for all proteins was 2.1%, with 14 of them increasing and 12 decreasing. There were eight proteins with *P*-values <.05 regarding the change in value, three of them increased and the remaining five proteins decreased. In addition, the changed ratio in values of proteins according to parameter variation were compared by upregulated and downregulated groups (Fig. 6B). As smoothness increased and the interaction effect accounted for a larger portion of the combined effect, the values of upregulated and downregulated proteins increased and decreased at higher rates, respectively. However, the magnitude of change was much larger for upregulated proteins than for downregulated proteins.

Next, we compared the significance of differences in protein values for each dementia subtype group indicated by the independent effect and the combined effect, denoted as *P*_group|*X*_ and *P*_group|*Z*_, respectively (Fig. 6C). The comparison results indicate that the *P*-values for protein values of the combined effect was lower on average than those of the independent effect, and that 19 proteins were changed by GPN to values allowing for more clear distinction between group differences. Furthermore, as biomarkers where *P*_group|*Z*_ is smaller than *P*_group|*X*_ were denoted by discriminative proteins, their frequencies according to parameter variation were compared. Overall, the frequency was high for the smoothness between 10^−2^ and 10^1^ and the combining ratio between 0.3 and 0.6, and the highest frequency observed when 𝜇 was 1 with θ between 0.3 and 0.5.

#### Explanatory analysis

**Figure 7:**
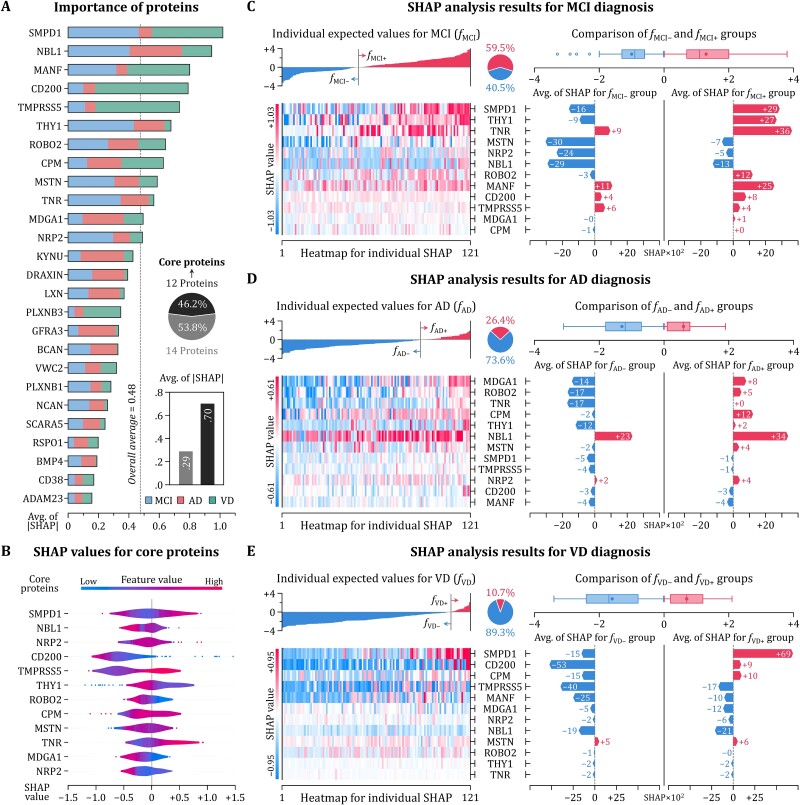
Explanatory analysis results for GPN interpretation. The importance of plasma protein biomarkers for diagnosing dementia subtypes was calculated by deriving SHAP values (A). Protein biomarkers with higher importance than the overall average were selected as core proteins, and the patterns of SHAP values were investigated (B). To identify effects of core proteins to the diagnosis, SHAP values were examined for each dementia subtype. By comparing individual expected values into positive and negative groups, the contribution of core proteins to the diagnosis of MCI, AD, and VD was clarified (C–E).

The combined effect of GPN was explained by SHAP values derived by applying XGboost classifier trained on the discovery cohort to the validation cohort. First of all, the importance of the biomarkers in GPN was measured (Fig. 7A). SMPD1 was revealed to be the most important protein, followed by NBL1 and MANF. Those three biomarkers were upregulated proteins: SMPD1 and MANF were VD-specific, and NBL1 was common to AD and VD. Then, we selected 12 biomarkers with absolute SHAP values exceeding the overall average as core proteins. Comparing the average of SHAP values for core proteins for all dementia subtypes (Fig. 7B), TNR showed the highest, followed by THY1 and SMPD1, and only these three proteins presented positive values due to the low proportion of AD and VD participants in the validation cohort. The remaining nine core proteins all indicated the average of SHAP values <0, with CD200 showing the lowest value.

In order to clarify the impact of core proteins on the diagnosis of each dementia subtype, SHAP values were analyzed by dividing them into subtypes, and the results include the individual expected value *f* and comparison of SHAP values between groups according to its positivity and negativity. First, there were 72 participants whose expected values for MCI were positive (*f*_MCI+_), ~60% of the validation cohort, and the remaining 49 participants showed *f*_MCI–_ (Fig. 7C). Through the heatmap for individual SHAP values, TNR and NBL1 showed the greatest influence on the increase and decrease in *f*_MCI_, respectively. Moreover, we compared the contributions of core proteins in *f*_MCI+_ and *f*_MCI–_ groups by calculating the group-wise average of SHAP values for each biomarker. SMPD1 and THY1 showed remarkable differences between two groups in that both proteins increased *f*_MCI+_ while decreasing *f*_MCI–_. Although TNR, MSTN, NRP2, and NBL1 also showed notable differences, they contributed to the common increase or decrease in both groups. Second, the individual expectations for AD were divided into 32 *f*_AD+_ and 89 *f*_AD–_ participants (Fig. 7D). NBL1 represented the noticeable individual SHAP value through the overall increase of the expected value for AD diagnosis. The group-wise comparison results indicated that MDGA1 and ROBO2 contribute the most to clarifying the differences between *f*_AD+_ and *f*_AD–_ groups. The effects of TNR and CPM were also significant, with both having a particular impact on decreasing *f*_AD–_ and increasing *f*_AD+_, respectively. Last, because the validation cohort included a small number of participants with VD, only 13 participants were included in the *f*_VD+_ group, while the remaining 108 were *f*_VD–_ (Fig. 7E). Therefrom, individual SHAP values of core proteins were generally <0, but SMPD1, CD200, and CPM contributed significantly to increasing the expected value for the *f*_VD+_ group. By simultaneously decreasing the expectations in the *f*_VD–_ group, those three proteins played a key role in clarifying the differences between groups.

## Discussion

In this study, dementia subtype diagnosis based on plasma protein biomarkers was performed by a two-stage approach. First, by comparing the MCI group with the AD or VD groups, 26 plasma proteins with significant differences in expression were selected as biomarkers. Our findings included well-known dementia-associated protein biomarkers, such as BMP4, CD200, MANF, PLXNB1, PLXNB3, and SMPD1 for AD [54–59] and BCAN, NCAN, and THY1 for VD [60, 61], as well as uncovering novel proteins associated with AD or VD. Subsequent analysis confirmed that the identified biomarkers were functionally related to the nervous system. Next, the identified plasma protein biomarkers were applied to the proposed model GPN for dementia subtype diagnosis. The most pronouncing feature of GPN is that the independent effect is propagated on the PPI network, and therefrom, the interactive effect between proteins is extracted. This process enables the GPN to reflect global range of protein interactions, equivalent to the infinite order of PPIs. The predicted outcomes are derived after combining the independent and interactive effects. Experimental results on the Korean cohort presented that the differences between diagnostic groups were more remarkable in the combined effect and that the performance of GPN was better than existing methods by 10.4% on average. We also showed that the combined effect can be used for any classifier by improving the original performance. Furthermore, the contribution of biomarkers to the diagnostic results of each dementia subtype was confirmed, and therefrom, several proteins were identified as key factors for diagnosing MCI, AD, and VD.

**Figure 8:**
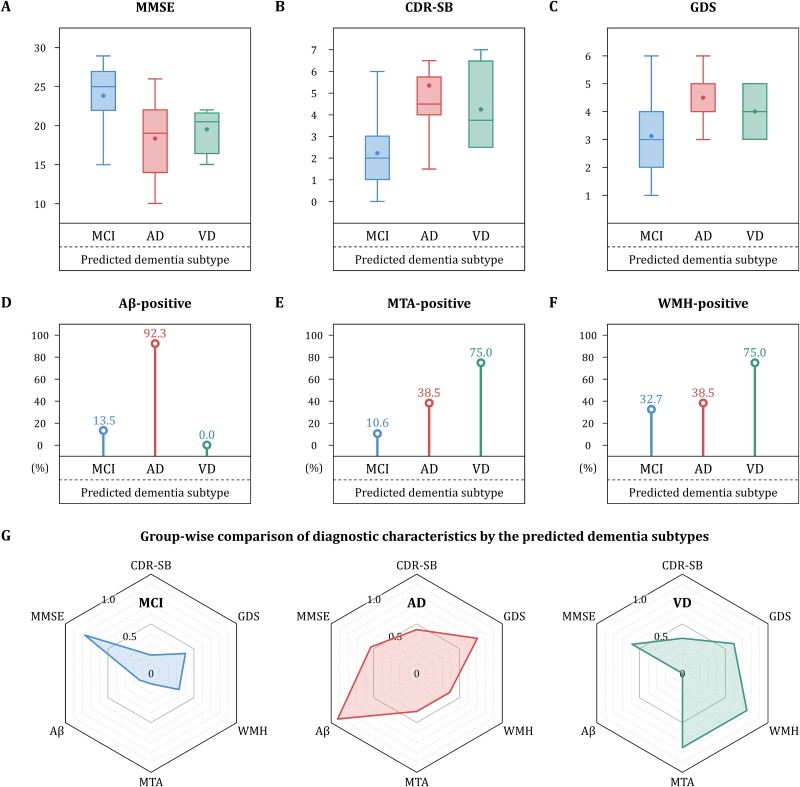
Comparison of clinical and diagnostic characteristics based on the predicted outcomes by GPN. Dementia subtype diagnosis results for the validation cohort were compared with clinically important test items: MMSE (A), CDR-SB (B), and GDS (C). The predicted outcomes were additionally compared with neuroimaging-based diagnostic biomarkers: Aβ deposition (D), neurodegeneration (E), and vascular neuropathology (F). By combining the comparison results, patterns for dementia subtypes were derived in terms of clinical characteristics and neuroimaging biomarkers (G).

At last, we investigated the utility of GPN in a real-world clinical setting by assessing its compatibility with key medical factors for diagnosing dementia subtypes (Supplementary Table S7). First, the predicted dementia subtypes were compared to clinically important test items: MMSE, CDR-SB, and GDS (Fig. 8A–C, respectively). The AD-predicted and VD-predicted groups showed lower cognitive function than the MCI-predicted group, and the degree of deterioration in the AD-predicted group was the highest. Next, our prediction results were compared to neuroimaging-based biomarkers: amyloid-β deposition (Aβ), neurodegeneration (MTA), and vascular neuropathology (WMH) (Fig. 8D–F, respectively). The positivity rates for Aβ and WMH were highest for the AD-predicted and VD-predicted groups, respectively, with the lowest MTA positive rate for the MCI-predicted group. Finally, the comprehensive patterns for each dementia subtype were derived by integrating comparison results (Fig. 8G). In sum, the predicted outcomes showed high compatibility compatible with the key medical factors, suggesting that the GPN may be a clinically useful tool for diagnosing dementia subtypes.

Here are some remarks on the method we proposed. First, a more precise diagnosis of dementia subtypes through the GPN is achievable by assaying a larger number of plasma proteins. Our findings are limited to proteins in the Olink Neurology panel. Although the proteins belonging to this panel may be strongly associated with dementia, it is plausible that significant biomarkers could also be discovered in other panels focusing on inflammation, cell regulation, and metabolism. Therefore, employing additional protein assay panels provides greater scope for advancements in diagnosing dementia subtypes based on plasma protein biomarkers. Second, the GPN can be further advanced through the optimization of protein interactions. In this study, the GPN utilized the combined score of PPI, which simply multiplied seven types of interaction scores. However, the contribution of each type to the diagnosis of dementia subtypes varies. Furthermore, the impact of interaction will be different for each protein. Therefore, the GPN will become more technically sophisticated by optimizing interaction types and differentiating the impact of interactions for each protein. Third, the real-world applicability of GPN for routine patient screening can be enhanced by incorporating neuroimaging-based diagnostic markers. Although the proposed method predicts the diagnosis of AD and VD by clinicians with high accuracy, there may be interclinician variability in the medical opinions regarding patients’ conditions. Accordingly, the use of GPN for neuroimaging-based outcomes that can be objectively judged may improve its real-world applicability in clinical settings. Additionally, developing a panel of plasma protein biomarkers specific to neuroimaging-based dementia subtypes and a GPN-based software that learns from the data generated could be of great clinical value. Therefore, as a follow-up study, we aim to extend the application of GPN to the prediction of neuroimaging-based diagnostic markers to improve its clinical practicality. Finally, GPN is scalable to various domains where interactions between entities significantly affect the target outcome. In those domains, such as chemical interaction, brain connectivity, genomic polymorphism [62], and social network, a wide range of interactions need to be considered in that there will be chained diffusion arise from synergistic effects by entity interactions. The global awareness of interactions in GPN will be beneficial for tasks in those domains. Moreover, as the experiment suggests, the propagation process in GPN can benefit any classifier. Therefore, leveraging interactive effects will be even more useful.

**Key Points**

- Plasma protein biomarkers have been considered as promising tools in diagnosing dementia subtypes and sophisticated by utilizing machine learning (ML).
- Although various graph convolutional networks can alleviate the limitation of previous ML-based studies that exclude interactions between proteins, they still have issues originated by their local awareness.
- The proposed method extracts the globally interactive effect between proteins that propagates the independent effect on the protein–protein interaction (PPI) network.
- The global awareness for PPI in our model significantly contributes to improve performance of dementia subtype diagnosis compared to the existing methods.

## Supplementary Material

Supplement_bbae428

## Data Availability

The data used in this study are available upon request from the BICWALZS consortium biobank (http://www.bicwalzs.com) as well as the National Biobank of Korea (https://biobank.nih.go.kr) which is a central biobank supporting for the Korea Biobank Project. Conflict of interest: None declared.
