# Supplementary material for: Identification of molecular subtypes of dementia by using blood-proteins interaction-aware graph propagational network
Source: Brief Bioinform. 2024 Sep 3;25(5):bbae428. doi: 10.1093/bib/bbae428 (PMC11370639; doi:10.1093/bib/bbae428)
Supplement: Supplement_bbae428 [file supplement_bbae428.docx]

Supplementary Data

Identification of molecular subtypes of dementia by using

blood proteins interaction aware graph propagational network

Sunghong Park^1^, Chang Hyung Hong^2^, Sang Joon Son^2^, Hyun Woong Roh^2^, Doyoon Kim^1,3^,

Hyunjung Shin^4,5,*^, and Hyun Goo Woo^1,5,6,*^

^1^ Department of Physiology, Ajou University School of Medicine, Suwon, 16499, Republic of Korea

^2^ Department of Psychiatry, Ajou University School of Medicine, Suwon, 16499, Republic of Korea

^3^ Department of Biomedical Science, Graduate School, Ajou University, Suwon, 16499, Republic of Korea

^4^ Department of Industrial Engineering, Ajou University, Suwon, 16499, Republic of Korea

^5^ Department of Artificial Intelligence, Ajou University, Suwon, 16499, Republic of Korea

^6^ Ajou Translational Omics Center (ATOC), Research Institute for Innovative Medicine, Ajou University Medical Center, Suwon, 16499, Republic of Korea

**^*^ Corresponding authors:** Hyunjung Shin (shin@ajou.ac.kr) and Hyun Goo Woo (hg@ajou.ac.kr)

| Contents | |
| --- | --- |
| Supplementary Table S1. | Comparison results for demographic and clinical characteristics in discovery cohort |
| Supplementary Table S2. | Comparison results for demographic and clinical characteristics in validation cohort |
| Supplementary Table S3. | Differentially expressed proteins for Alzheimer’s disease |
| Supplementary Table S4. | Differentially expressed proteins for vascular dementia |
| Supplementary Table S5. | Comparison results for expression levels of identified plasma protein biomarkers |
| Supplementary Table S6. | Gene Ontology analysis results for identified protein biomarkers |
| Supplementary Table S7. | Comparison results for clinical and diagnostic characteristics by predicted outcomes |

**Supplementary Table S1.** Comparison results for demographic and clinical characteristics in discovery cohort

| **Characteristics** | **Dementia subtypes** | | | *P*-value |
| --- | --- | --- | --- | --- |
|  | MCI (*n* = 191) | AD (*n* = 58) | VD (*n* = 22) |  |
| Age, median (IQR), yr | 73 (66–77) | 74 (68–78) | 72 (68–76) | 0.712 |
| Female, No. (%) | 138 (72.3) | 39 (67.2) | 15 (68.2) | 0.733 |
| MADRS, median (IQR) | 15 (7–24) | 10 (4–20) | 20 (10–28) | 0.058 |
| MMSE, median (IQR) | 26 (23–27) | 20 (16–23) | 20 (15–23) | < 0.001 |
| CDR-SB, median (IQR) | 2.0 (1.0–2.5) | 5.0 (4.0–6.0) | 5.0 (3.0–6.0) | < 0.001 |
| GDS, median (IQR) | 3 (2–3) | 4 (4–5) | 4 (4–5) | < 0.001 |
| APOE genotype, No. (%) |  |  |  |  |
| *ε*2 allele carrier | 32 (16.8) | 1 (1.7) | 5 (22.7) | 0.007 |
| *ε*4 allele carrier | 38 (19.9) | 29 (50.0) | 6 (27.3) | < 0.001 |
| Aβ-positive, No. (%) | 41 (21.5) | 49 (84.5) | 1 (4.5) | < 0.001 |
| MTA-positive^†^, No. (%) | 24 (12.6) | 33 (57.9) | 13 (59.1) | < 0.001 |
| WMH-positive^‡^, No. (%) | 63 (33.2) | 21 (36.8) | 20 (90.9) | < 0.001 |

Abbreviations: IQR, interquartile range; MADRS, Montgomery-Asberg depression rating scale; MMSE, mini-mental status examination; CDR-SB, clinical dementia rating sum of boxes; GDS, global deterioration scale; APOE, apolipoprotein E; Aβ, amyloid beta; MTA, medial temporal lobe atrophy score; WMH, white matter hyperintensity.

^†^ MTA scale is divided into left and right and subdivided into 0 to 4 according to severity, and in this study, MTA-positive was set for cases where the sum of left and right sides was 5 or more.

^‡^ WMH scale is divided into three types (mild, moderate, and severe), and WMH-positive was set for moderate and severe.

**Supplementary Table S2.** Comparison results for demographic and clinical characteristics in validation cohort

| **Characteristics** | **Dementia subtypes** | | | *P*-value |
| --- | --- | --- | --- | --- |
|  | MCI (*n* = 98) | AD (*n* = 15) | VD (*n* = 8) |  |
| Age, median (IQR), yr | 72 (66–77) | 76 (70–79) | 77 (76–78) | 0.021 |
| Female, No. (%) | 69 (70.4) | 11 (73.3) | 7 (87.5) | 0.583 |
| MADRS, median (IQR) | 15 (6–24) | 4 (3–17) | 24 (18–30) | 0.008 |
| MMSE, median (IQR) | 25 (22–27) | 17 (15–21) | 20 (18–22) | < 0.001 |
| CDR-SB, median (IQR) | 2.0 (1.0–2.5) | 5.0 (4.0–5.0) | 4.0 (3.0–6.0) | < 0.001 |
| GDS, median (IQR) | 3 (2–3) | 4 (4–5) | 5 (4–5) | < 0.001 |
| APOE genotype, No. (%) |  |  |  |  |
| *ε*2 allele carrier | 14 (14.3) | 0 (0.0) | 1 (12.5) | 0.298 |
| *ε*4 allele carrier | 23 (23.5) | 10 (66.7) | 0 (0.0) | < 0.001 |
| Aβ-positive, No. (%) | 12 (12.2) | 14 (93.3) | 0 (0.0) | < 0.001 |
| MTA-positive^†^, No. (%) | 6 (6.1) | 7 (46.7) | 6 (75.0) | < 0.001 |
| WMH-positive^‡^, No. (%) | 28 (28.6) | 6 (40.0) | 8 (100.0) | < 0.001 |

Abbreviations: IQR, interquartile range; MADRS, Montgomery-Asberg depression rating scale; MMSE, mini-mental status examination; CDR-SB, clinical dementia rating sum of boxes; GDS, global deterioration scale; APOE, apolipoprotein E; Aβ, amyloid beta; MTA, medial temporal lobe atrophy score; WMH, white matter hyperintensity.

^†^ MTA scale is divided into left and right and subdivided into 0 to 4 according to severity, and in this study, MTA-positive was set for cases where the sum of left and right sides was 5 or more.

^‡^ WMH scale is divided into three types (mild, moderate, and severe), and WMH-positive was set for moderate and severe.

**Supplementary Table S3.** Differentially expressed proteins for Alzheimer’s disease

| **Symbol** | **Ensemble Protein ID** | **Mean expression values** | | **Group-wise comparison** | | |
| --- | --- | --- | --- | --- | --- | --- |
|  |  | **Case**  (*N* = 58) | **Control**  (*N* = 191) | **Mean difference**  (Case – Control) | **–log_10_(*P*-value)** | **Type** |
| LXN | ENSP00000264265 | 2.96 | 2.25 | 0.71 | 4.09 | Up-reg. |
| PLXNB1 | ENSP00000351338 | 4.07 | 2.79 | 1.28 | 3.51 | Up-reg. |
| BMP4 | ENSP00000245451 | 5.66 | 5.02 | 0.64 | 3.17 | Up-reg. |
| NRP2 | ENSP00000353582 | 8.59 | 8.51 | 0.08 | 1.56 | Up-reg. |
| NBL1 | ENSP00000289749 | 5.89 | 5.78 | 0.11 | 1.55 | Up-reg. |
| NCAN | ENSP00000252575 | 8.81 | 9.05 | -0.25 | 3.08 | Down-reg. |
| DRAXIN | ENSP00000294485 | 3.66 | 4.06 | -0.40 | 3.03 | Down-reg. |
| ROBO2 | ENSP00000417335 | 5.33 | 5.49 | -0.16 | 2.90 | Down-reg. |
| BCAN | ENSP00000331210 | 4.87 | 5.09 | -0.22 | 2.35 | Down-reg. |
| KYNU | ENSP00000264170 | 8.59 | 8.99 | -0.41 | 2.17 | Down-reg. |
| THY1 | ENSP00000284240 | 9.97 | 10.11 | -0.14 | 2.17 | Down-reg. |
| MDGA1 | ENSP00000402584 | 5.25 | 5.52 | -0.27 | 2.00 | Down-reg. |
| RSPO1 | ENSP00000348944 | 3.26 | 3.54 | -0.28 | 1.93 | Down-reg. |
| CD38 | ENSP00000226279 | 5.25 | 5.53 | -0.28 | 1.79 | Down-reg. |
| GFRA3 | ENSP00000274721 | 4.96 | 5.10 | -0.15 | 1.77 | Down-reg. |
| CPM | ENSP00000448517 | 8.00 | 8.10 | -0.10 | 1.56 | Down-reg. |
| ADAM23 | ENSP00000264377 | 4.86 | 5.03 | -0.16 | 1.54 | Down-reg. |
| TNR | ENSP00000356646 | 3.10 | 3.24 | -0.14 | 1.49 | Down-reg. |
| CD200 | ENSP00000420298 | 6.17 | 6.27 | -0.10 | 1.46 | Down-reg. |
| MSTN | ENSP00000260950 | 3.15 | 3.32 | -0.17 | 1.41 | Down-reg. |
| VWC2 | ENSP00000341819 | 5.78 | 5.98 | -0.20 | 1.40 | Down-reg. |
| SCARA5 | ENSP00000346990 | 8.49 | 8.61 | -0.12 | 1.33 | Down-reg. |

**Supplementary Table S4.** Differentially expressed proteins for vascular dementia

| **Symbol** | **Ensemble Protein ID** | **Mean expression values** | | **Group-wise comparison** | | |
| --- | --- | --- | --- | --- | --- | --- |
|  |  | **Case**  (*N* = 22) | **Control**  (*N* = 191) | **Mean difference**  (Case – Control) | **–log_10_(*P*-value)** | **Type** |
| PLXNB3 | ENSP00000442736 | 4.69 | 4.27 | 0.43 | 2.61 | Up-reg. |
| MANF | ENSP00000432799 | 8.63 | 8.12 | 0.52 | 2.44 | Up-reg. |
| LXN | ENSP00000264265 | 2.93 | 2.25 | 0.68 | 2.33 | Up-reg. |
| TMPRSS5 | ENSP00000299882 | 3.52 | 3.27 | 0.26 | 1.80 | Up-reg. |
| PLXNB1 | ENSP00000351338 | 3.95 | 2.79 | 1.17 | 1.74 | Up-reg. |
| BMP4 | ENSP00000245451 | 5.65 | 5.02 | 0.63 | 1.72 | Up-reg. |
| SMPD1 | ENSP00000340409 | 5.66 | 5.45 | 0.21 | 1.45 | Up-reg. |
| NBL1 | ENSP00000289749 | 5.92 | 5.78 | 0.14 | 1.31 | Up-reg. |
| CD200 | ENSP00000420298 | 6.11 | 6.27 | -0.16 | 1.53 | Down-reg. |

**Supplementary Table S5.** Comparison results for expression levels of identified plasma protein biomarkers

| **Symbol** | **Biomarker** | | **Expression level by dementia subtypes** | | | **Comparison by dementia subtypes** | | |
| --- | --- | --- | --- | --- | --- | --- | --- | --- |
|  | AD | VD | MCI | AD | VD | AD **–** MCI | VD **–** MCI | AD **–** VD |
| DRAXIN | Down | - | 4.06 | 3.66 | 3.78 | **–**9.83 | **–**6.81 | **–**3.35 |
| RSPO1 | Down | - | 3.54 | 3.26 | 3.28 | **–**7.95 | **–**7.50 | **–**0.49 |
| MSTN | Down | - | 3.32 | 3.15 | 3.13 | **–**5.03 | **–**5.73 | **+**0.75 |
| CD38 | Down | - | 5.53 | 5.25 | 5.37 | **–**5.01 | **–**2.88 | **–**2.25 |
| TNR | Down | - | 3.24 | 3.10 | 3.13 | **–**4.35 | **–**3.30 | **–**1.09 |
| BCAN | Down | - | 5.09 | 4.87 | 4.93 | **–**4.27 | **–**3.22 | **–**1.10 |
| MDGA1 | Down | - | 5.52 | 5.25 | 5.38 | **–**4.92 | **–**2.55 | **–**2.49 |
| KYNU | Down | - | 8.99 | 8.59 | 8.73 | **–**4.52 | **–**2.91 | **–**1.69 |
| ADAM23 | Down | - | 5.03 | 4.86 | 4.83 | **–**3.23 | **–**3.82 | **+**0.60 |
| VWC2 | Down | - | 5.98 | 5.78 | 5.82 | **–**3.34 | **–**2.77 | **–**0.59 |
| GFRA3 | Down | - | 5.10 | 4.96 | 5.04 | **–**2.92 | **–**1.36 | **–**1.60 |
| ROBO2 | Down | - | 5.49 | 5.33 | 5.41 | **–**2.89 | **–**1.31 | **–**1.63 |
| CD200 | Down | Down | 6.27 | 6.17 | 6.11 | **–**1.66 | **–**2.51 | **+**0.86 |
| NCAN | Down | - | 9.05 | 8.81 | 8.95 | **–**2.72 | **–**1.12 | **–**1.64 |
| THY1 | Down | - | 10.11 | 9.97 | 10.00 | **–**1.39 | **–**1.14 | **–**0.25 |
| SCARA5 | Down | - | 8.61 | 8.49 | 8.64 | **–**1.34 | **+**0.39 | **–**1.75 |
| CPM | Down | - | 8.10 | 8.00 | 8.14 | **–**1.23 | **+**0.53 | **–**1.78 |
| NRP2 | Up | - | 8.51 | 8.59 | 8.60 | **+**0.90 | **+**1.07 | **–**0.16 |
| NBL1 | Up | Up | 5.78 | 5.89 | 5.92 | **+**1.96 | **+**2.42 | **–**0.46 |
| SMPD1 | - | Up | 5.45 | 5.52 | 5.66 | **+**1.28 | **+**3.88 | **–**2.56 |
| TMPRSS5 | - | Up | 3.27 | 3.29 | 3.52 | **+**0.65 | **+**7.91 | **–**7.22 |
| MANF | - | Up | 8.12 | 8.35 | 8.63 | **+**2.79 | **+**6.35 | **–**3.46 |
| PLXNB3 | - | Up | 4.27 | 4.41 | 4.69 | **+**3.38 | **+**9.96 | **–**6.37 |
| BMP4 | Up | Up | 5.02 | 5.66 | 5.65 | **+**12.72 | **+**12.58 | **+**0.12 |
| LXN | Up | Up | 2.25 | 2.96 | 2.93 | **+**31.57 | **+**30.22 | **+**1.03 |
| PLXNB1 | Up | Up | 2.79 | 4.07 | 3.95 | **+**46.00 | **+**41.90 | **+**2.81 |

**Supplementary Table S6.** Gene Ontology analysis results for identified protein biomarkers

| **(A) Major GO terms for identified protein biomarkers** | | | |
| --- | --- | --- | --- |
| **GO term ID** | **Description** | **–log_10_(*Q*-value)** | **Frequency (%)** |
| GO:0005576 | Extracellular region^*^ | 5.4 | 21 (80.8) |
| GO:0022008 | Neurogenesis^†^ | 5.3 | 14 (53.8) |
| GO:0007399 | Nervous system development^†^ | 5.1 | 16 (61.5) |
| GO:0030182 | Neuron differentiation^†^ | 4.5 | 12 (46.2) |
| GO:0048699 | Generation of neurons^†^ | 4.4 | 12 (46.2) |
| GO:0045595 | Regulation of cell differentiation^†^ | 3.5 | 12 (46.2) |
| GO:0051128 | Regulation of cellular component organization^†^ | 2.9 | 13 (50.0) |
| GO:0005615 | Extracellular space^*^ | 2.6 | 15 (57.7) |
| GO:0007166 | Cell surface receptor signaling pathway^*^ | 2.1 | 11 (42.3) |
| GO:0048468 | Cell development^*^ | 1.9 | 11 (42.3) |
| GO:0023052 | Signaling^*^ | 1.3 | 15 (57.7) |
| **(B) Major GO terms for total assayed proteins** | | | |
| **GO term ID** | **Description** | **–log_10_(*Q*-value)** | **Frequency (%)** |
| GO:0005576 | Extracellular region^*^ | 12.0 | 57 (65.5) |
| GO:0007166 | Cell surface receptor signaling pathway^*^ | 11.3 | 39 (44.8) |
| GO:0048468 | Cell development^*^ | 8.6 | 36 (41.4) |
| GO:0005615 | Extracellular space^*^ | 6.3 | 42 (48.3) |
| GO:0007154 | Cell communication^‡^ | 5.8 | 49 (56.3) |
| GO:0023052 | Signaling^*^ | 5.5 | 48 (55.2) |
| GO:0007165 | Signal transduction^‡^ | 5.4 | 46 (52.9) |
| GO:0023051 | Regulation of signaling^‡^ | 5.4 | 39 (44.8) |
| GO:0010646 | Regulation of cell communication^‡^ | 5.4 | 39 (44.8) |
| GO:0009966 | Regulation of signal transduction^‡^ | 5.2 | 36 (41.4) |

^*^ GO terms common to the identified protein biomarkers and total assayed proteins

^†^ GO terms specific to the identified protein biomarkers

^‡^ GO terms specific to the total assayed proteins

**Supplementary Table S7.** Comparison results for clinical and diagnostic characteristics by predicted outcomes

| **Characteristics** | **Predicted outcomes** | | | *P*-value |
| --- | --- | --- | --- | --- |
|  | MCI (*n* = 104) | AD (*n* = 13) | VD (*n* = 4) |  |
| MMSE, median (IQR) | 25 (22–27) | 19 (14–22) | 21 (18–22) | < 0.001 |
| CDR-SB, median (IQR) | 2.0 (1.0–3.0) | 4.5 (4.0–5.5) | 4.0 (2.5–6.0) | < 0.001 |
| GDS, median (IQR) | 3 (2–3) | 4 (4–5) | 4 (3–5) | < 0.001 |
| Aβ-positive, No. (%) | 14 (13.5) | 12 (92.3) | 0 (0.0) | < 0.001 |
| MTA-positive^†^, No. (%) | 11 (10.6) | 5 (38.5) | 3 (75.0) | < 0.001 |
| WMH-positive^‡^, No. (%) | 34 (32.7) | 5 (38.5) | 3 (75.0) | 0.213 |

Abbreviations: IQR, interquartile range; MMSE, mini-mental status examination; CDR-SB, clinical dementia rating sum of boxes; GDS, global deterioration scale; Aβ, amyloid beta; MTA, medial temporal lobe atrophy score; WMH, white matter hyperintensity.

^†^ MTA scale is divided into left and right and subdivided into 0 to 4 according to severity, and in this study, MTA-positive was set for cases where the sum of left and right sides was 5 or more.

^‡^ WMH scale is divided into three types (mild, moderate, and severe), and WMH-positive was set for moderate and severe.
